# A diagnostic dilemma in breast pathology – benign fibroadenoma with multinucleated stromal giant cells

**DOI:** 10.1186/1746-1596-3-33

**Published:** 2008-08-01

**Authors:** Helen M Heneghan, Sean T Martin, Mary Casey, Igdam Tobbia, Fadel Benani, Kevin M Barry

**Affiliations:** 1 Dept. of Surgery, Mayo General Hospital, Ireland; 2 Dept. of Radiology, Mayo General Hospital, Ireland; 3 Dept. of Pathology, Mayo General Hospital, Ireland

## Abstract

Fibroadenomas are common benign breast tumours that display a characteristic pathological morphology, although several epithelial and stromal variations exist. A very rare histological finding is the presence of multinucleated giant cells throughout the stroma of a benign fibroadenoma. Cells of this type, which are more commonly found incidentally within the interlobular stroma of breast tissue, are benign and should not be mistaken for malignant cells on microscopic examination. Unfortunately a lack of awareness of this pathological entity can lead to diagnostic confusion amongst pathologists resulting in the multinucleate giant cells being mistaken for highly mitotic cells and consequently the fibroadenoma being mistaken for a malignant lesion. This may have serious implications for the subsequent management of the patient. The presence of this unusual cell type in the stroma does not alter the prognosis of otherwise benign lesion. We encountered two such cases at our institution in a six month period recently. We present their histories along with relevant radiological, microscopic and immunohistochemical features, followed by a discussion of this unusual pathological entity.

## Case Presentation

**Figure 1:**
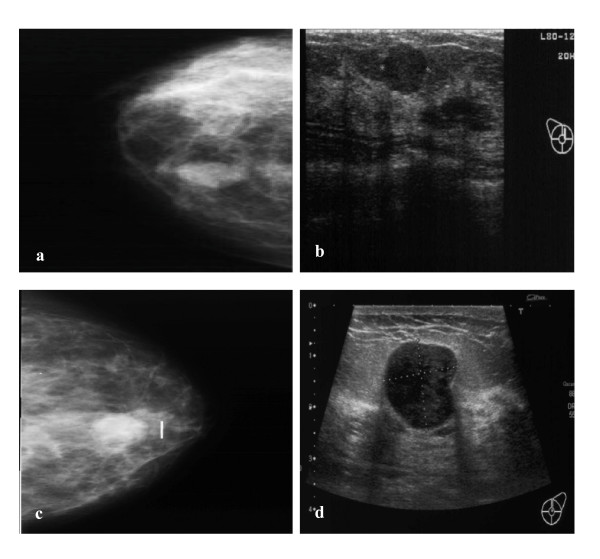
**Radiology images from 2 cases presenting with a breast mass**. a. Case 1. Mammogram of right breast. b. Case 1. Ultrasound of right breast. c. Case 2. Mammogram of left breast. d. Case 2. Ultrasound of left breast.

**Figure 2:**
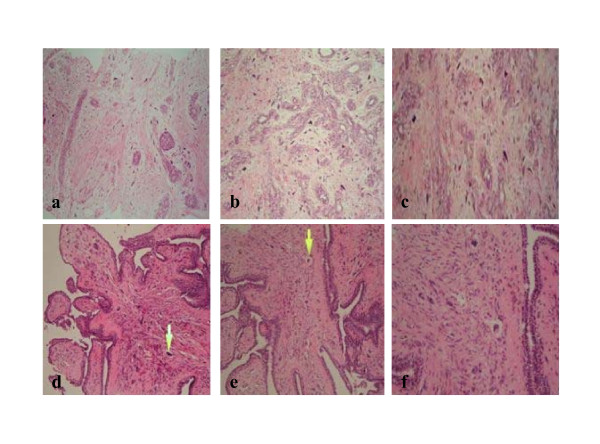
**Microscopy of breast core biopsies**. a. Case 1 (H&E stain, × 200). b. Case 1 (H&E stain, × 200). c. Case 1 (H&E stain, × 400). d. Case 2 (H&E stain, × 200). e. Case 2 (H&E stain, × 200). f. Case 2 (H&E stain, × 400).

**Figure 3:**
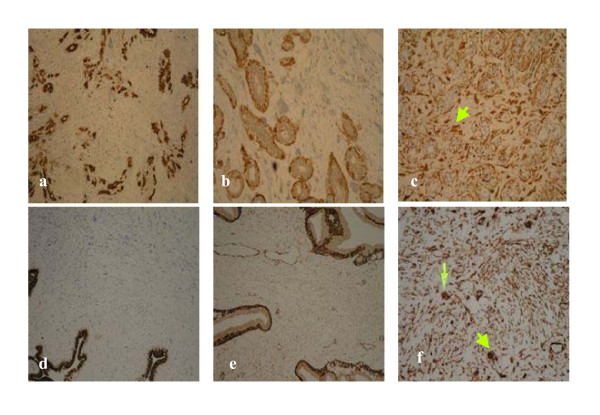
**Immunohistochemical stains on core breast biopsy tissue**. a. Case 1: Pancytokeratin. b. Case 1: SMA. c. Case 1: Vimentin (arrow marks multinucleated giant cells in stroma). d. Case 2: Pancytokeratin. e. Case 2: SMA. f. Case 2: Vimentin (arrow marks multinucleated giant cells in stroma).

A 42 year old female was referred to the Breast Clinic for assessment of a palpable right breast lump. She had detected the breast lump six weeks previously during routine self examination and did not complain of any mastalgia, nipple discharge, skin changes or systemic symptoms. She had no personal or family history of breast cancer and had never used the oral contraceptive pill (OCP) or hormone replacement therapy. Clinical examination revealed a non-tender, mobile 2 cm solid mass in the upper outer quadrant of the right breast. Mammography and Ultrasonography confirmed the presence of a 2 cm solid mass in the right upper quadrant (Figures 1a, 1b). Core biopsy demonstrated fibroadipose tissue with stromal calcification. Given the clinical and pathological findings the patient opted for surgical excision of the lesion. Gross examination of the specimen revealed a well circumscribed firm nodule measuring 2.5 × 2.0 cm. The cut surface was firm and tan-gray in colour, with a whorled appearance. Microscopically the tumour shows a benign epithelial component with elongated, branching ducts and cellular stroma. The stroma was composed of cells with giant nuclei some of which are multi-nucleated. Mitosis of these cells was not seen (Figures 2a, 2b, 2c). The stromal cells stained negative for the Estrogen and Progesterone receptors (ER, PR respectively) Pancytokeratin (AE1/3 & CAM 5.2), Muscle Specific Actin, S100 and desmin, and stained positive for Vimentin; a general mesenchymal marker and suggestive of cells of myofibroblastic origin (Figures 3a, 3b, 3c). The conclusive diagnosis was that of a fully excised benign fibroadenoma, with multinucleated giant cells throughout its stroma. She made an uneventful postoperative recovery and follow-up has shown no recurrence of the lesion.

The second case is that of a 48 year old lady referred to the Breast Clinic with a two month history of a left breast lump and mastalgia. She denied nipple discharge, nipple inversion or skin changes. She had no relevant past medical history, had never used the OCP and had no family history of breast cancer. On examination, a 1.5 cm tender solid mass was palpable in the upper inner quadrant of the left breast. Ultrasonography revealed the presence of a number of small benign cysts with a single solid lobulated mass lesion at 12 o'clock measuring 17 mm in diameter (Figure 1c). Mammography confirmed the presence of a smooth mass measuring 2 cm in diameter in the retro-areolar region of left breast (Figure 1d).

Ultrasound guided tru-cut biopsy was performed. Histological analysis demonstrated cores and fragments of fibroadenomatous breast tissue, with numerous uniformly giant and multi-nucleated cells intermingled with fibroblasts throughout the stroma, (Figures 2d, 2e, 2f).

Immunohistochemistry staining of these giant cells was negative again for ER & PR status, pancytokeratin (AE1/3 & CAM 5.2) and muscle specific actin (Fig 3d, 3e) as well as for S100 protein and desmin. They showed only positivity with vimentin (Figures 3f). No malignant changes were seen and a diagnosis of benign fibroadenoma of the left breast with a multi-nucleated giant cell stroma was made. The patient declined surgical excision of the lesion.

## Discussion

In 1979 Rosen first described the presence of Multinucleated Stromal Giant Cells (MSGCs) in the breast, as an incidental finding in breast specimens from 14 patients with breast carcinoma [1]. The tissue foci containing these atypical MSGCs were located in otherwise normal areas of the mammary gland and were usually distinct from the carcinomata. Rosen concluded that these cells represented a non-neoplastic and possibly reparative process. Subsequently MSGCs have been described in several other breast lesions raising an interesting differential diagnosis, mainly with benign disorders but also on occasion in association with malignant lesions [2-5]. MSGCs similar to those occurring in breast tissue have also been found to occur in the vagina, uterine cervix, nasal polyps, urinary bladder epithelium, anus, and in lesions of the oral cavity. Their presence in other benign tumours has also been described, including pleomorphic lipomas, leiomyomas and fibromas, schwannomas, and in variants of dermatofibromas with atypical cells. The pathogenesis of MSGCs in breast tissue and in the lower female genital tract is unclear. Rosen postulated that the MSGCs in mammary tissue may have been related to alterations in hormone levels during the perimenopausal period. Indeed all 14 patients in whom these cells were found in Rosen's case series, were aged between 40–50 years. It has also been noted that MSGCs in the female genital tract are associated with pregnancy and exogenous progestins.

### Diagnostic pitfalls

Due to its rarity, few cases of MSGCs in benign breast tumours have been described cytologically. Hence recognition and correct interpretation of their presence is difficult, yet crucial to forming an accurate diagnosis. Incorrect interpretation of these unusual cells as malignant cells can lead to misdiagnosis of more sinister conditions, such as malignant phyllodes tumor and metaplastic carcinoma. Consequently treatment of a lesion bearing MSGCs could potentially be misguided. Another diagnostic pitfall to be aware of upon identifying multinucleated giant cells in breast lesions is their resemblance histologically to osteoclast-like giant cells which are also infrequently reported to occur in a similar spectrum of both benign and malignant breast lesions [6-9]. A distinction must be made between these two cell types as it has diagnostic and prognostic implications. In fact osteoclast-like giant cells in association with malignant breast epithelial cells is indicative of a mammary carcinoma with postulated poor prognosis. Several reports have noted a less favourable outcome for patients with mammary carcinomas containing osteoclast-like giant cells when compared with conventional ductal adenocarcinoma [9].

In a review article on this subject in 2004, Cai states that it is the combination of osteoclast-like giant cells and cancer cells in association, which allows the diagnosis of this rare type of adenocarcinoma, even from a fine needle aspiration specimen [8]. However in scanty biopsy specimens, the bland-appearing multinucleated cells may be misinterpreted simply as foreign-body giant cells such as that seen in necrosis of fat.

Therefore Cai cautions that it is the cytologic features of the breast epithelial cells, rather than the presence of the osteoclast-like giant cells in isolation, upon which the diagnosis of mammary carcinoma with osteoclast-like giant cells is based on. Thus, a careful search for malignant epithelial cells should always be performed upon recognition of any osteoclast-like giant cells in a breast biopsy, to avoid a false negative diagnosis [10]. Several cytological differences are noted between MSGCs and osteoclast-like giant cells to help in their differentiation. Multinucleated stromal cells have multiple overlapping nuclei and scant cytoplasm, and they are usually located within the stroma, with no association with the epithelial cells [11]. In contrast, osteoclast-like giant cells have over 20 centrally located nuclei, and are found primarily in the advancing edges of the infiltrating tumour mass or in the lumen of the cribriform epithelial ducts, and in fine needle aspiration smears the osteoclast-like giant cells are intimately admixed with malignant epithelial cells [12]. In phyllodes tumors for example, which are uncommon fibroepithelial neoplasms with a prominent stromal component and where MSGCs may be seen on rare occasions, these particular multinucleated stromal cells are different from osteoclast-like giant cells, showing a linear nuclear arrangement or floret-like pattern [3,6,13,14].

The immunohistochemical staining pattern is also different for MSGCs and osteoclast-like giant cells. Tse described the immunohistochemical profile of the infrequent MSGCs in phyllodes tumours, commenting that both the MSGCs and stromal cells expressed vimentin strongly but not desmin; and in some but not all fibroepithelial tumours, both MSGCs and stromal cells expressed actin weakly. These features suggest that MSGCs may be off myofibroblastic differentiation [3].

Indeed both of our cases stained strongly positive for vimentin though negative for actin, indicating likely fibroblastic origin. On the other hand, osteoclast-like giant cells have an immunohistochemistry profile typical of histiocytic differentiation, staining positive for the typical osteoclast markers, CD68, CD1a, tartrate-resistant acid phosphatase (TRAP), and negative for cytokeratin, epithelial membrane antigen, lysozyme, and estrogen and progesterone receptors. Their staining pattern thus rejects any epithelial, endothelial or trophoblastic origin of these cells [15].

### Histological features and origin of MSGCs

The histogenesis of MSGCs remains obscure and controversial however, with several conflicting reports in the literature [1,3,4]. As briefly mentioned above, the most consistent finding with immunohistochemical studies is that MSGCs stain strongly positive for Vimentin, which is one of the Type II 1F proteins in the cytoskeleton and is known to play a significant role in maintaining cell shape, integrity of the cytoplasm, and stabilizing cytoskeletal interactions [16]. Histopathological reports indicate that cells showing Vimentin positivity is broadly suggestive of a myofibroblastic origin [17]. Abd el-All, who reported on the histopathological and immunohistochemical features of breast spindle cell tumours, then suggests that the Vimentin/CD34 positive fibroblast of mammary stroma could be the result of differentiation from a pluripotential mesenchymal precursor cell with the potential to differentiate toward several mesenchymal lines [18]. MSGCs also show inconsistent focal positive staining with histiocytic markers such as alpha-1-antitrypsin, alpha-1-antichymotrypsin, HAM-56, CD34, and CD68. These markers of histiocytic differentiation are more consistently found positive in osteoclast-like giant cells. As shown in our two cases MSGCs stain negatively with immunoperoxidase stains for oestrogen and progesterone receptors, cytokeratins (AE 1/3 and CAM 5.2), S100 protein, muscle specific actin and desmin, further supporting their fibroblastic origin.

### Differential Diagnosis

In our two cases had the presence of the bizarre multinucleated giant cells within the stroma of the benign breast fibroepithelial tumour been mistaken as evidence of malignancy then this could have led to a misdiagnosis, perhaps that of a malignant Phyllodes tumour (malignant cystosarcoma phyllodes) as described briefly above, or other breast carcinoma. MSGCs are similarly a rare finding in Breast carcinomas [10,19]. In a recent review of literature published by Cai G regarding the presence specifically of osteoclast-like multinucleated giant cells in mammary carcinomas [10], the occurrence of MSGCs in a variety of malignant tumours was also described. This report noted their presence in a spectrum of invasive and in-situ breast carcinomas and referred to their original description in malignant breast lesions by Rosen [1]. Notably, Rosen also commented on the diagnostic importance and rarity of MSGCs found in mammary carcinomas in his book 'Rosen's Breast Pathology' [11]. The bizarre multinucleated giant cells in invasive breast carcinoma are usually located within the stroma, with no relation to the epithelial cells. In this setting these MSGCs are distinguishable from osteoclast-like giant cells also by their pleomorphism and their distinct immunoprofile, as alluded to above and further described by Gupta [20].

The differential diagnosis upon recognition of MSGCs in breast lesions also includes Breast spindle cell tumours (BSCTs), and other hamartomas of the breast. BSCTs are a heterogeneous group including benign and malignant lesions, with different therapeutic and prognostic implications. Cytological examination followed by immunohistochemistry staining is critical to differentiate BSCTs from other breast lesions as well as to categorise the individual subtypes of spindle cell tumour. An accurate diagnosis is essential in order to appropriately manage the lesion and predict the prognosis for the patient [18]. For example with regard to myoepithelial tumours which are a subtype of BSCT composed of a dominant to pure population of myoepithelial cells, immunohistochemistry stains typically show positivity for actin, S-100, cytokeratin, p63 and CD10, all of which are consistent with a myoepithelial origin. These may be differentiated from fibroepithelial tumours on the basis that the spindle cells stain negative for CD34. Differentiation of myoepitheliomas into benign and malignant lesions is based on further cytologic features. Those features observed only in malignant myoepithelial lesions include pleomorphism, coarse nuclear chromatin, prominent nucleoli, high mitotic activity and tumour necrosis [21].

Hamartomas are breast lesions with varying amounts of benign epithelial elements, fibrous tissue, and fat and in rare cases the occurrence of giant cells has been noted. They lack a distinctive pathological appearance however most authors agree on a general characteristic pattern of interlobular fibrosis, which is defined as the presence of lobules within a fibrotic stroma, which surrounds and extends to between individual lobules and obliterates the usual interlobular specialised loose stroma [22]. Unfortunately however this pattern is not unique to hamartomas. Other commonly described yet nonetheless inconsistent features of hamartomas include the presence of varying proportions of pseudo-angiomatous stroma, and adipose tissue within the stroma [23]. Epithelial changes such as hyperplasia without atypia, cystic change, apocrine metaplasia, and adenosis have been described in a smaller proportion of hamartomas [23,24], as have other rare features including microcalcification, myoid differentiation, stromal oedema, and stromal giant cells. Furthermore, there are occasional case reports of coincidental in-situ, or invasive ductal or lobular carcinoma occurring in hamartomas [25]. Hence the correct identification of hamartomas is important because there are the problems of recurrence and coincidental epithelial malignancy. However diagnosis is difficult, and not reliant solely on any one histopathological technique or immunohistochemistry stain, due to the presence of the various elements. A complete triple assessment of these lesions is warranted to formulate the correct diagnosis, including correlation of the clinical impression with the radiologically distinct imaging findings and the above pathological features.

## Conclusion

The presence of these pleomorphic, multinucleated large cells in small specimens such as those obtained at fine needle aspiration or core biopsy, may be misinterpreted as a malignant process. The typical benign cytoarchitecture of a fibroadenomas, in a background of naked multiple nuclei indicates the benign nature of the lesion and has no known malignant potential. Correct recognition and the ability to differentiate these cells from malignant cells are dependent on a combination of conventional diagnostic pathological techniques: H&E and IHC staining using a small panel of antibodies. Awareness of this phenomenon is critical in facilitating accurate diagnosis and appropriate management of the patient.

## Competing interests

The authors declare that they have no competing interests.

## Authors' contributions

HH is primarily responsible for drafting, literature search, submission and revising the manuscript. SM was involved in drafting and revising the manuscript, FB and IT evaluated the immunohistochemical stainings and confirmed the diagnoses, FB also was involved in revising the manuscript, MC performed and interpreted the radiological investigations for the patients. KB performed the surgeries, supplied relevant clinical information about the patients and directed management of the patients clinically. All authors read and approved the final manuscript.

## Authors’ consent to Publication (copyright)

I Helen Heneghan, the Corresponding Author have the right to grant on behalf of all authors of this manuscript, and do grant on behalf of all authors, an exclusive licence (or non-exclusive for government employees) on a worldwide basis to the BMJ Publishing Group Ltd and its Licensees to permit this article (if accepted) to be published in JCP and any other BMJPGL products to exploit all subsidiary rights, as set out in your licence.
